# Validating Risk Prediction Models for Multiple Primaries and Competing Cancer Outcomes in Families With Li-Fraumeni Syndrome Using Clinically Ascertained Data

**DOI:** 10.1200/JCO.23.01926

**Published:** 2024-04-03

**Authors:** Nam H. Nguyen, Elissa B. Dodd-Eaton, Jessica L. Corredor, Jacynda Woodman-Ross, Sierra Green, Angelica M. Gutierrez, Banu K. Arun, Wenyi Wang

**Affiliations:** ^1^ The University of Texas MD Anderson Cancer Center, Department of Bioinformatics and Computation Biology, Houston, TX; ^2^ Rice University, Department of Statistics, Houston, TX; ^3^ The University of Texas MD Anderson Cancer Center, Department of Clinical Cancer Genetics, Houston, TX; ^4^ The University of Texas MD Anderson Cancer Center, Department of Breast Medical Oncology, Houston, TX

## Abstract

**PURPOSE:**

There exists a barrier between developing and disseminating risk prediction models in clinical settings. We hypothesize that this barrier may be lifted by demonstrating the utility of these models using incomplete data that are collected in real clinical sessions, as compared with the commonly used research cohorts that are meticulously collected.

**MATERIALS AND METHODS:**

Genetic counselors (GCs) collect family history when patients (ie, probands) come to MD Anderson Cancer Center for risk assessment of Li-Fraumeni syndrome, a genetic disorder characterized by deleterious germline mutations in the *TP53* gene. Our clinical counseling-based (CCB) cohort consists of 3,297 individuals across 124 families (522 cases of single primary cancer and 125 cases of multiple primary cancers). We applied our software suite LFSPRO to make risk predictions and assessed performance in discrimination using AUC and in calibration using observed/expected (O/E) ratio.

**RESULTS:**

For prediction of deleterious *TP53* mutations, we achieved an AUC of 0.78 (95% CI, 0.71 to 0.85) and an O/E ratio of 1.66 (95% CI, 1.53 to 1.80). Using the LFSPRO.MPC model to predict the onset of the second cancer, we obtained an AUC of 0.70 (95% CI, 0.58 to 0.82). Using the LFSPRO.CS model to predict the onset of different cancer types as the first primary, we achieved AUCs between 0.70 and 0.83 for sarcoma, breast cancer, or other cancers combined.

**CONCLUSION:**

We describe a study that fills in the critical gap in knowledge for the utility of risk prediction models. Using a CCB cohort, our previously validated models have demonstrated good performance and outperformed the standard clinical criteria. Our study suggests that better risk counseling may be achieved by GCs using these already-developed mathematical models.

**Figure:**
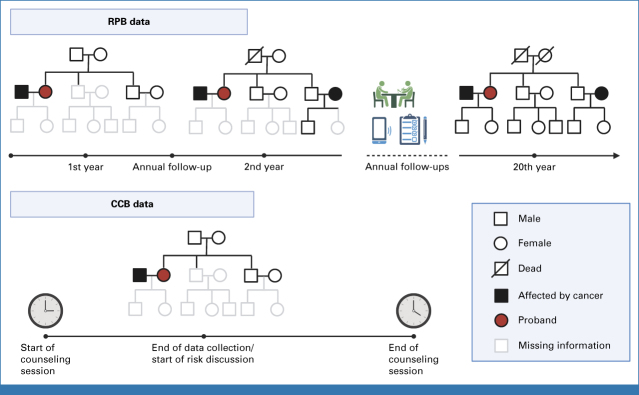

## INTRODUCTION

Li-Fraumeni syndrome (LFS) is a hereditary cancer syndrome identified by deleterious germline mutations in the *TP53* tumor suppressor gene.^1^ Patients with LFS are at significantly increased risks of many cancer types.^1-3^ The lifetime risks are 93% and 73% for women and men, respectively,^4^ with a 50% risk of second primary malignancy for cancer.^5^ Conversations with patients regarding genetic testing and cancer screening have been challenging, partly because genetic counselors (GCs) could only provide general, as compared with personalized, cancer risks associated with LFS.^6^ Risk prediction models have been developed for other hereditary cancer syndromes, such as the hereditary breast and ovarian cancer syndrome and its associated genes BRCA1/2. Among those, the Tyrer-Cuzick,^7^ BRCAPRO,^8^ and CanRisk^9,10^ models are used in the current National Comprehensive Cancer Network (NCCN) guidelines (version 4.2024) to facilitate recommendation of at-risk individuals for testing of breast cancer susceptibility genes. LFS, however, remained an untouched area until recently. We developed two models for families with LFS: (1) a competing-risk model that predicts cancer-specific (CS) risks for the first primary^11^ and (2) a recurrent event model that extends the prediction to multiple primary cancer (MPC).^12^ These models were trained on an LFS cohort rich in family history, and successfully validated on independent cohorts.^13,14^

**CONTEXT**

**Key Objective**
Using a patient cohort that was collected in real genetic counseling sessions, we perform a validation study to expedite the clinical utility of already-developed risk prediction models.
**Knowledge Generated**
Despite the frequent missing information in our clinical counseling-based patient cohort, the risk prediction models, which were trained and validated on carefully collected research-based data sets, outperform the clinical criteria when predicting deleterious germline *TP53* mutations for untested patients. For predictions of cancer risks, our models achieve performances that are comparable with the previous validation studies using research cohorts in most prediction objectives.
**Relevance *(R.G. Maki)***
A novel model helps better predict risk of cancer development in patients with Li-Fraumeni syndrome. These types of models, along with primary cancer screening, will hopefully improve the care for patients with familial cancer syndromes.*
*Relevance section written by *JCO* Associate Editor Robert G. Maki, MD, PhD, FACP, FASCO.

**FIG 1.**
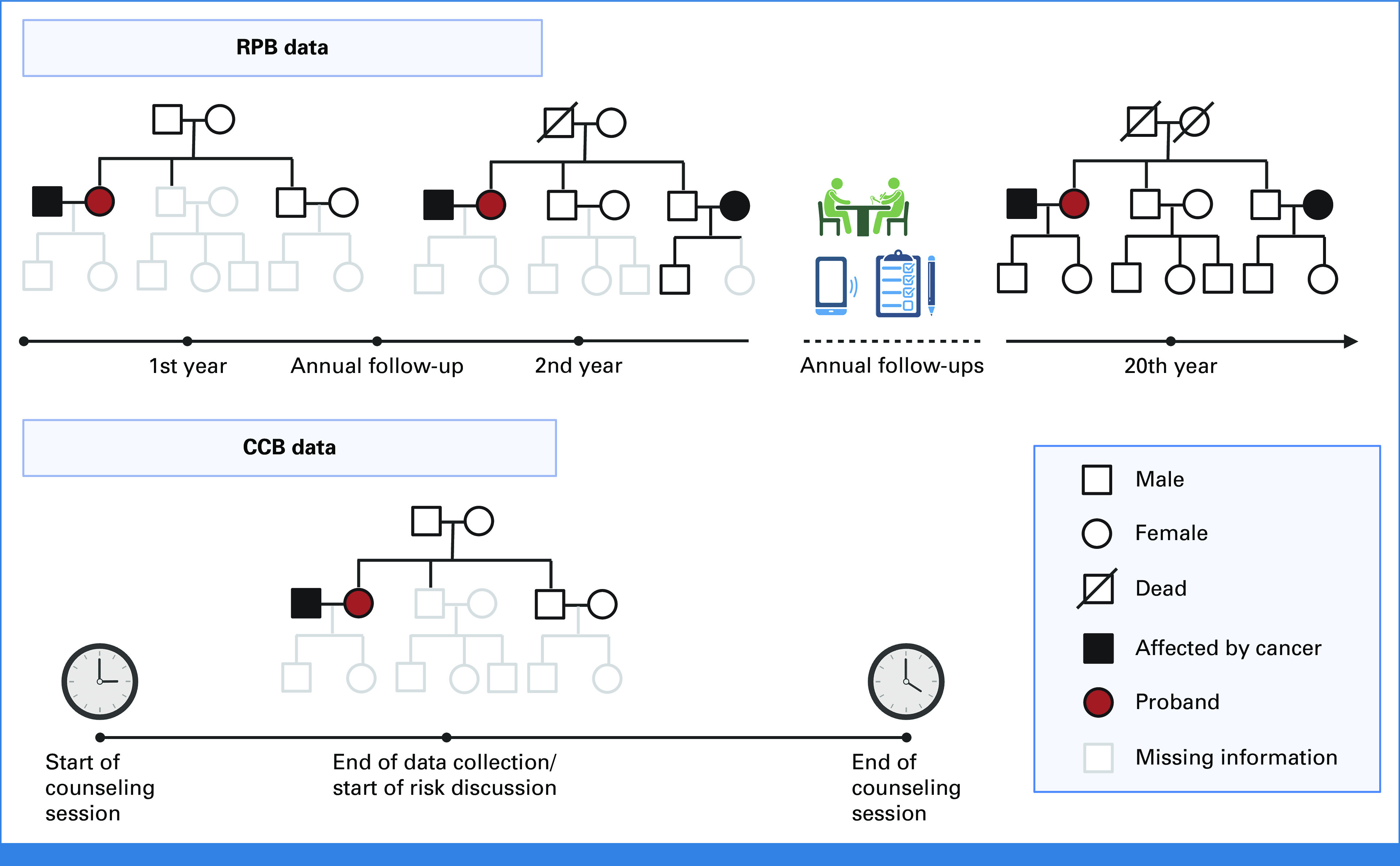
Comparison of the data collection process for RPB and CCB cohorts. RPB data are collected and updated over an extended period of time to ensure completeness and accuracy for research purposes, whereas CCB data represent a snapshot of information taken by genetic counselors over approximately 20 minutes during 1-hour counseling sessions. CCB, clinical counseling-based; RPB, research protocol-based.

The data sets used to train and validate these risk prediction models were research protocol-based (RPB). RPB refers to data that are collected via rigorous procedures to obtain complete and accurate patient cohorts for research purposes (Fig 1). Study investigators contact eligible patients for data collection via extensive use of questionnaires and phone interviews. Follow-ups are conducted regularly to add data and to acquire new incidences of cancer diagnoses, the latest births or deaths within the family, and any additional germline testing information. This diligent data collection process could go on for 20-30 years.^15-17^ RPB data sets are ideal for training statistical models to estimate key epidemiological parameters of a study population.

RPB data, however, do not represent data sets that are typically observed and collected in clinical settings, where patients with cancer history that is indicative of an inherited syndrome make appointments with medical professionals for in-depth risk assessment. We use the term clinical counseling-based (CCB) to refer to the data that are encountered by GCs during counseling sessions (Fig 1). CCB differs significantly from RPB because patients may not have accurate and complete family histories and some families have younger members who have not developed cancer. This leads to a higher rate of missing information such as family relationship, age of death, and age at cancer diagnoses. On the basis of this snapshot of family history (collected over approximately 20 minutes), GCs perform a comprehensive risk assessment, communicate these risks with the patients, and potentially recommend at-risk individuals for further testing and screening.

Owing to these wide discrepancies in data quality, it is important to determine whether statistical models that are trained and validated on RPB cohorts can perform well enough on a CCB cohort to be clinically useful. Given the large number of risk prediction models for hereditary cancer syndromes,^7,8,10,18,19^ it is surprising to see very few that made into the clinics.^20^ One potential reason is these models were mostly validated using well-established research databases or registry data^21-26^ rather than clinical data.^27^ In this study, we validate our risk prediction models on a CCB cohort of 124 families whose probands underwent genetic counseling at the Clinical Cancer Genetics (CCG) program at MD Anderson Cancer Center (MDACC) between 2000 and 2020.

## MATERIALS AND METHODS

### Patient Cohorts

Using a collection of 189 families that were recruited through probands diagnosed with pediatric sarcoma at MDACC from 1944 to 1982,^15-17^ we have estimated the model parameters for risk prediction.^11,12^ We refer the readers to the Data Supplement (online only) for detailed descriptions of this data set.

The validation data set was separately collected on *TP53* mutation carriers from the CCG program at MDACC. Personal and family history were collected during a genetic counseling session and immediately entered into the patient's electronic medical record. Data were automatically pulled into a Progeny database used by the CCG program for tracking families. This database includes patients counseled between year 2000 and 2020. For this study, only patients who were identified to have a pathogenic or likely pathogenic germline mutation in *TP53* through single-gene testing or multigene panel were included. Patients who did not meet the Classic^3^ or Chompret^28,29^ criteria were tested either because of clinical suspicion from a certified GC or they were identified on panel testing performed on suspicion for other hereditary cancer syndromes. Testing was performed in several Clinical Laboratory Improvement Amendments/College of American Pathologists-certified laboratories. Family members of the confirmed *TP53* mutation carrier were not required to undergo additional testing; however, recommendations for family member testing were made during standard-of-care genetic counseling sessions. This cohort includes a total of 3,297 individuals from 124 families. Summaries of both data sets are given in Table 1.

**TABLE 1.** Categorization of All Family Members in the Research Cohort (189 families) Used as Training Data and the Clinical Cohort (124 families) Used as Validation Data by Sex, No. of Primary Cancers and Mutation Status

Characteristics	Research Cohort (training data)				Clinical Cohort (validation data)			
	WT	Mut	U	Total	WT	Mut	U	Total
Male, No.								
Healthy	295	9	1,276	1,580	17	13	1,376	1,406
SPC	105	25	139	269	3	15	210	228
MPC	3	14	8	25	1	10	20	31
Subtotal	403	48	1,423	1,874	21	38	1,606	1,665
Female, No.								
Healthy	341	8	1,207	1,546	21	20	1,203	1,244
SPC	120	21	102	243	3	33	260	296
MPC	4	19	10	33	1	59	32	92
Subtotal	465	48	1,319	1,832	25	112	1,495	1,632
Total	868	96	2,742	3,706	46	150	3,101	3,297

Abbreviations: MPC, multiple primary cancer; Mut, *TP53* mutation; SPC, single primary cancer; U, unknown; WT, wildtype.

### Risk Prediction Models

We previously developed and validated two models for LFS risk predictions^11-14^ using RPB data. The CS model^11^ estimates the CS age-at-onset penetrance, defined as the probability of developing a particular cancer type before all others by a certain age given the patient's covariates and cancer history. We consider three competing cancer types: (1) sarcoma, including soft-tissue and osteosarcoma, (2) breast cancer, and (3) all other cancer types combined. We also include death as another competing risk. The hazard function of each cancer type is modeled via frailty modeling^30^ and depends on patient-specific covariates $X={\left\{G,S,G\times S\right\}}^{T}$, where G denotes the *TP53* mutation status (mutation or wildtype) and S denotes the sex (male or female). We compute a family-wise likelihood using the peeling algorithm,^31^ followed by ascertainment bias correction,^32^ and finally estimate the regression coefficients via Markov chain Monte Carlo. The age-at-onset penetrance at age t for the k-th cancer type, denoted by $q_{k}^{cs}\left(t|X\right)$, can then be computed from the estimated model parameters.

We further developed the MPC model^12^ to estimate the MPC-specific age-at-onset penetrance, defined as the probability of developing the next primary cancer by a certain age given the patient's covariates and cancer history. We model the occurrence process of cancer using a nonhomogenous Poisson process to capture the age dependency of cancer risks over a patient's lifetime.^33,34^ The intensity function of this Poisson process follows frailty modeling as before and depends on patient-specific covariates $X\left(t\right)={\{G,S,G\times S,D\left(t\right),G\times D\left(t\right)\}}^{T}$, where we introduce D(t), an indicator variable for whether a patient has developed a primary cancer before time t, to allow the risks of subsequent primary cancers to depend on the first.^35-37^ Following a similar estimation procedure, we compute $q_{l}^{mpc}\left(t_{l}|t_{l-1},X\left(t_{l-1}\right)\right)$, the age-at-onset penetrance at age $t_{l}$ for the l-th primary cancer conditional on the previous cancer at age $t_{l-1}$. We only estimate penetrances up to the second primary because of limited occurrences of the third primary and beyond.

Most patients do not undergo genetic testing (ie, G is unknown). Both models use the BayesMendel method^38^ to infer the probability of carrying a deleterious *TP53* variant for these patients on the basis of their family history. We provide the detailed computations of this probability in the Data Supplement. The cancer risks for untested patients are then given by weighted sums of the corresponding penetrances for each genotype status, with weights being the probabilities of mutation and wildtype. We refer the readers to the study by Shin et al^11,12^ for the full technical details of the two models.

### Validation Study Design

We excluded family members who had either (1) unknown age at cancer diagnoses for the first or second primary cancer or (2) unknown age at last contact if they had never had cancer, or both, from the set of validation subjects. Missing information among the excluded family members can still negatively affect performance on the validation subjects because the key assumption of our models lies in the Mendelian inheritance pattern that is implicitly demonstrated by cancer outcomes within the family.^39^

We first validated our models' ability to predict an individual's probability of carrying a deleterious *TP53* mutation given the provided family history. We used the models to make predictions for the validation subjects, including the probands, that had undergone genetic testing, and then compared the predicted outcomes with the confirmed genotypes. In the calculations, we disregarded all testing results. This mimicked a real scenario, in which GCs use the models to assess the risks of the probands, and to identify at-risk individuals within their families. We then conducted a similar validation, in which we made the predictions for nonproband family members given the probands' confirmed genotypes, to evaluate the impact of this additional information.

Next, we ran the models to make cancer risk predictions. We further excluded the probands because of ascertainment bias. For the MPC model, we divided the validation subjects into three groups: those without cancer (group 1), those with single primary cancer (SPC; group 2), and those with MPC (group 3). We then validated the model in two tasks: (1) to predict individuals with at least one primary cancer versus those without and (2) to predict individuals with MPC versus those with SPC. For the first task, we recorded the age at last contact for group 1 and the age at first cancer diagnosis for groups 2 and 3. For each individual, we computed the risk probability to develop a first primary cancer at the recorded age $t_{1}$. By varying the cutoff on the risk estimates and comparing the predictions with the actual outcomes, we constructed the receiver operating characteristic curve (ROC) and calculated the AUC. For comparison, we also used the Kaplan-Meier (KM) method to achieve the same prediction objective. Specifically, we estimated the KM-based penetrance for each combination of sex (male or female) and carrier status (mutation or wildtype). Given an individual's covariates, we computed the risk probability at age $t_{1}$ as a weighted sum of the corresponding KM-based penetrance estimates in a similar manner as the MPC model. For the second task, we recorded the age at last contact for group 2 and the age at second cancer diagnosis for group 3. We computed the risk probability to develop a second primary at the recorded age $t_{2}$ given the covariates and cancer history up to age $t_{1}$. We similarly constructed the ROC curve as described above.

For the CS model, we recorded the age at first event (ie, the age at diagnosis of the first primary if the individual had a cancer history or the age at last contact if otherwise). We used the model to compute the risk probability at the recorded age $t_{1}$ for each of the four competing outcomes (ie, sarcoma, breast cancer, other cancer types, and mortality). We constructed ROC curves for predicting one cancer type versus all other outcomes.

In addition to AUCs, which measures discrimination between binary outcomes, we also assessed calibration via the observed/expected (O/E) ratios. The 95% CIs for the performance metrics were computed via bootstrapping.

## RESULTS

### Comparison of Clinical and Research Data

Our training data set, being RPB, was collected via rigorous research protocols to obtain complete information for research purposes. By contrast, the CCG data set, being CCB, represented snapshots of information taken by GCs during counseling sessions. Table 2 highlights the main differences, most notably the level of missing data between RPB and CCB based on the key summary statistics of these two data sets (all comparisons presented a Chi-square test with *P* < .001).

**TABLE 2.** Comparison of a Research Cohort (pediatric sarcoma as training data) and a Clinical Cohort (clinical cancer genetics as validation data) on the Extent of Missing Age at Last Contact and Missing Age at Cancer Diagnoses at Both Family and Individual Levels

Summary Statistics	RPB Training Data	CCB Validation Data
No. of families		
All family members, No. (%)		
Complete data	189 (100)	10 (8)
Missing age at last contact only	0 (0)	46 (37)
Missing age at cancer diagnosis only	0 (0)	0 (0)
Missing both age at last contact and age at cancer diagnosis	0 (0)	68 (55)
Total	189	124
Chi-square test	*P* < .001	
First-degree relatives and spouse only, No. (%)		
Complete data	189 (100)	68 (55)
Missing age at last contact only	0 (0)	41 (33)
Missing age at cancer diagnosis only	0 (0)	10 (8)
Missing both age at last contact and age at cancer diagnosis	0 (0)	5 (4)
Total	189	124
Chi-square test	*P* < .001	
No. of individuals		
All family members, No. (%)		
Complete data	3,706 (100)	1,748 (53)
Missing age at last contact only	0 (0)	1,339 (41)
Missing age at cancer diagnosis only	0 (0)	138 (4)
Missing both age at last contact and age at cancer diagnosis	0 (0)	72 (2)
Total	3,706	3,297
Chi-square test	*P* < .001	
First-degree relatives and spouse only, No. (%)		
Complete data	1,126 (100)	487 (79)
Missing age at last contact only	0 (0)	105 (17)
Missing age at cancer diagnosis only	0 (0)	19 (3)
Missing both age at last contact and age at cancer diagnosis	0 (0)	2 (0.3)
Total	1,126	613
Chi-square test	*P* < .001	
No. of individuals per family		
Min	3	1
5th percentile	4	1
10th percentile	5	4
25th percentile	6	16
Median	7	27
Mean	20	27
75th percentile	10	36
90th percentile	15	48
95th percentile	72	54
Max	719	75

NOTE. Summary statistics for the number of individuals per family are reported to contrast the depth of data collection procedures in research and clinical cohorts as they happen in the unit of families.

Abbreviations: CCB, clinical counseling-based; RPB, research protocol-based.

### Validation of *TP53* Mutation Prediction

**FIG 2.**
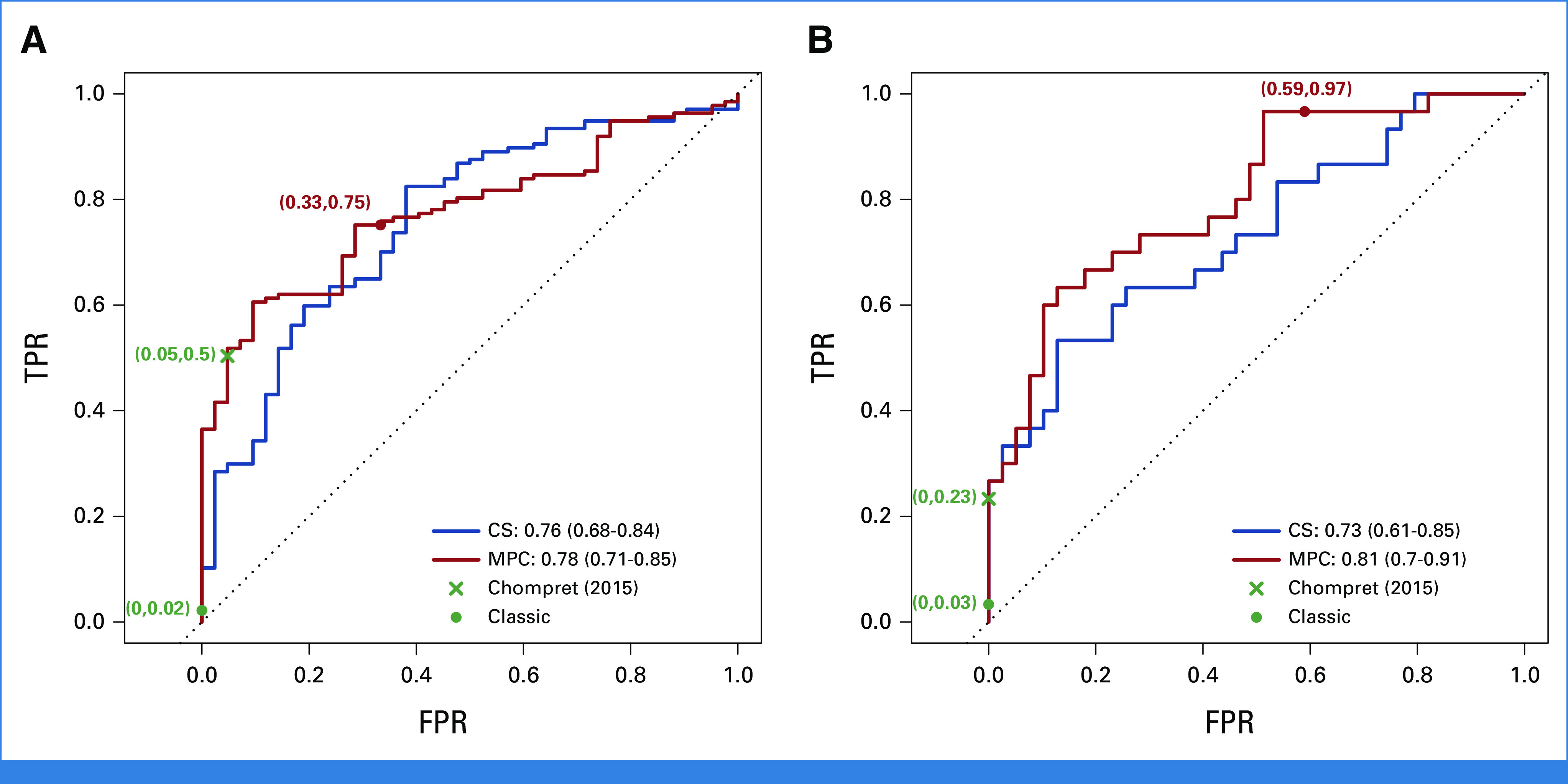
ROC curves and the 95% bootstrapped CIs of the AUCs for *TP53* mutation predictions in the CCG cohort using the CS and MPC models under two scenarios: (A) predict mutations for both the probands and their family members when no genotype information is available and (B) predict mutations for family members given the probands' confirmed genotypes. The TPRs and FPRs of the MPC model at cutoff probability of 0.20, as suggested by validation on research cohorts, are shown in both scenarios. The classic and Chompret criteria are shown for comparison. Sample sizes: (A) n (mutation carriers) = 137, n (wildtypes) = 42 and (B) n (mutation carriers) = 30, n (wildtypes) = 39. CCG, Clinical Cancer Genetics; CS, cancer-specific; FPR, false-positive rate; MPC, multiple primary cancer; ROC, receiver operating characteristic; TPR, true-positive rate.

In Figure 2A, we compared the models' predictions of *TP53* mutations with the Classic^3^ and Chompret^28,29^ criteria, which are recommended in the NCCN guidelines (version 2.2024) for LFS. Our CS and MPC models achieved AUCs of 0.76 (95% CI, 0.68 to 0.84) and 0.78 (95% CI, 0.71 to 0.85), respectively. With a decision threshold of 0.20 as recommended by our previous validation study using RPB cohorts,^39^ the MPC model achieved a true-positive rate (TPR) of 0.75 and a false-positive rate (FPR) of 0.33, whereas the Chompret criteria achieved a near-zero FPR at the cost of a low TPR. The MPC model achieved a much better O/E ratio of 1.66 (95% CI, 1.53 to 1.80) compared with the CS model, which showed underestimation with an O/E ratio of 7.83 (95% CI, 7.20 to 8.47). The MPC model performed better than the CS model in both criteria, thus providing further support for selecting the MPC model as default in our clinical risk prediction tool LFSPRO.^39^ Our validation study on research cohorts^39^ achieved AUCs near 0.85 and O/E ratios around 1.30 with the MPC model. Thus, the predictive performance on clinical data was indeed lower, but still at a reasonable level. Given the probands' confirmed genotypes, the MPC model obtained a slightly better AUC of 0.81 (95% CI, 0.70 to 0.91; Fig 2B). With a decision threshold of 0.20, we achieved a TPR of 0.97 and a FPR of 0.59 using the MPC model. The calibration of both models improved further, with almost perfect O/E ratios of 1.10 (95% CI, 0.80 to 1.39) and 0.96 (95% CI, 0.70 to 1.21) for CS and MPC, respectively. Under this perfect calibration, a new cutoff probability might be needed to attain a balanced trade-off between TPR and FPR. We note, however, that this scenario is less clinically relevant because carrier probabilities are considered most useful in pretest counseling of the family. Overall, these results highlight a strong advantage of our models over the standard criteria when using the available information.

### Validation of Cancer Risk Prediction

**FIG 3.**
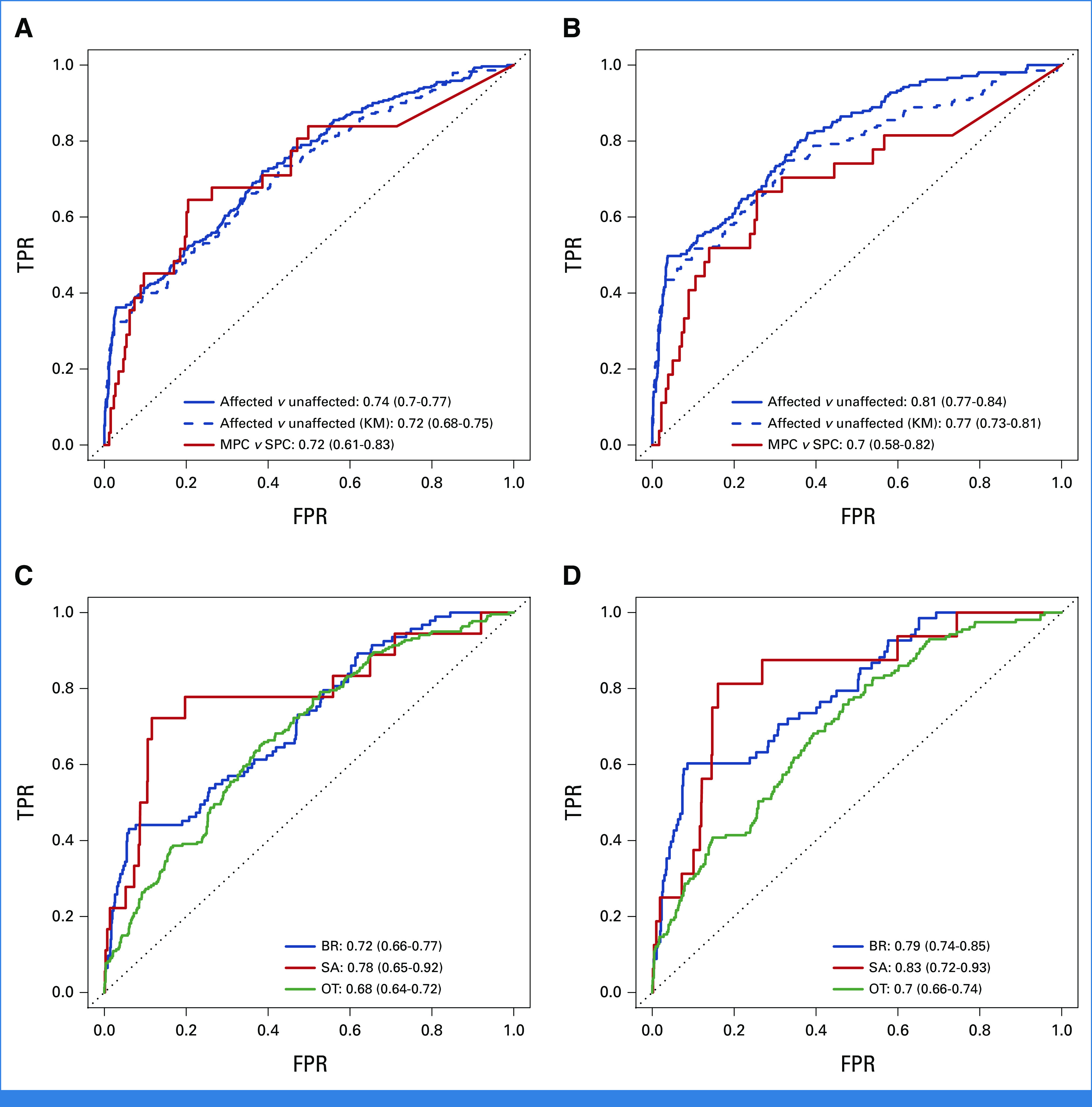
ROC curves and the 95% bootstrapped CIs of the AUCs for predictive performance of the CS and MPC models on the CCG cohort under two scenarios: (A) and (C) all validation subjects are included and (B) and (D) only known and inferred mutation carriers and wildtypes are included. For comparison, the KM method is used to predict at least one cancer versus no cancer. Sample sizes in scenario (A) and (C): n (unaffected) = 1,264, n (SPC) = 259, n (MPC) = 31, n (BR) = 94, n (SA) = 18, n (OT) = 220, n (D) = 497, n (A) = 879. Sample sizes in scenario (B) and (D): n (unaffected) = 907, n (SPC) = 180, n (MPC) = 27, n (BR) = 69, n (SA) = 16, n (OT) = 157, n (D) = 379, n (A) = 617. A, alive; BR, breast cancer; CCG, Clinical Cancer Genetics; CS, cancer-specific; D, death; FPR, false-positive rate; KM, Kaplan-Meier; MPC, multiple primary cancer; OT, all other cancer types combined; ROC, receiver operating characteristic; SA, sarcoma; SPC, single primary cancer; TPR, true-positive rate.

When discriminating between individuals with and without cancer, the MPC model achieved a slightly better performance than the KM method, with AUCs of 0.74 (95% CI, 0.70 to 0.77) versus 0.72 (95% CI, 0.68 to 0.75; Fig 3A). When predicting SPC versus MPC, it achieved an AUC of 0.72 (95% CI, 0.61 to 0.83; Fig 3A). This validation included subjects with unknown genotypes. In practice, given the large difference in risks between the two genotype groups, it would be more accurate to communicate the risk predictions after the patients have had confirmed testing results. Thus, we performed a secondary validation, which, in addition to tested individuals, included only those with mutation probabilities that were either >0.1 as inferred carriers or smaller than 0.001 as inferred wildtypes. Figure 3B shows improvement in performance, with the MPC model still outperforming the KM method. This performance was comparable with our previous validation study,^14^ which showed an AUC of 0.73 (95% CI, 0.67 to 0.79) when predicting cancer versus no cancer and an AUC of 0.77 (95% CI, 0.69 to 0.85) when predicting SPC versus MPC on a research cohort.

The CS model achieved AUCs of 0.72 (95% CI, 0.66 to 0.77), 0.78 (95% CI, 0.65 to 0.92), and 0.68 (95% CI, 0.64 to 0.72) for separately predicting each outcome versus all others (Fig 3C). These AUCs noticeably improved to 0.79 (95% CI, 0.74 to 0.85), 0.83 (95% CI, 0.72 to 0.93), and 0.70 (95% CI, 0.66 to 0.74), respectively, in the secondary validation (Fig 3D). Compared with validation on research cohorts,^13^ we obtained a higher AUC for sarcoma but lower AUCs for breast cancer and other cancer types. Sarcoma, however, was strongly underrepresented among the validation subjects as shown in Figures 3C and 3D, hence a larger sample size would be needed to make a meaningful comparison.

Finally, we observed that the calibration performances of both models were reasonably close to 1 and improved slightly in the secondary validation (Table 3).

**TABLE 3.** O/E Ratios, Along With the 95% CIs, for Various Prediction Objectives of the CS and MPC Models Under Two Scenarios: (1) All Validation Subjects are Included (yes) and (2) Only Known and Inferred Mutation Carriers and Wildtypes are Included (no)

Prediction Objective	MPC Model		
	All Validation Subjects	O/E Ratio	95% CI
At least one cancer *v* no cancer	No	1.42	1.24 to 1.59
At least one cancer *v* no cancer (KM)	No	0.65	0.57 to 0.72
SPC *v* MPC	No	1.23	0.80 to 1.66
At least one cancer *v* no cancer	Yes	1.59	1.42 to 1.75
At least one cancer *v* no cancer (KM)	Yes	0.67	0.60 to 0.74
SPC *v* MPC	Yes	1.26	0.84 to 1.68
	**CS Model**		
**Prediction Objective**	**All Validation Subjects**	**O/E Ratio**	**95% CI**
Breast cancer *v* all other outcomes	No	1.51	1.16 to 1.85
Sarcoma *v* all other outcomes	No	0.63	0.33 to 0.94
Other cancers *v* all other outcomes	No	1.39	1.19 to 1.60
Breast cancer *v* all other outcomes	Yes	1.75	1.40 to 2.09
Sarcoma *v* all other outcomes	Yes	0.59	0.32 to 0.87
Other cancers *v* all other outcomes	Yes	1.45	1.27 to 1.63

Abbreviations: CS, cancer-specific; KM, Kaplan-Meier; MPC, multiple primary cancer; O/E, observed/expected; SPC, single primary cancer.

## DISCUSSION

We successfully conducted a validation of our LFS risk prediction models using a unique CCB patient cohort collected at MDACC over 2000-2020. These models had been trained and validated on RPB data sets.^11-14^ Our study was carefully designed to mimic scenarios that GCs encounter in clinical settings, with 20%-45% missing data, hence, to our knowledge, was the first risk prediction validation study of its kind. Our CS and MPC models demonstrated excellent discrimination and good calibration when predicting deleterious germline *TP53* mutations. As expected, the performance was lower than the validation results obtained using RPB cohorts,^39^ most likely due to the lack of important data such as age at last contact and age at cancer diagnoses. For predictions of cancer risks, both models displayed performance that was comparable with previous validation studies on RPB cohorts^13,14^ in most aspects. Given the promising results, we have implemented our risk prediction models as a simple, interactive R/Shiny app^40^ for users without any programming background, to expedite clinical applications.

The performance of our models provides evidence that our research-based penetrance estimates can be accurately applied to clinical data sets that are routinely collected in counseling sessions. Our results further suggest that the models can serve as an alternative, or a complement, to the Chompret criteria, which are currently used by GCs for counseling. Finally, GCs can use our models to provide more tailored discussions on the basis of the personalized cancer risks of their patients. The good calibration performance further ensures that the risk estimates are consistent with the true probabilities, which would be useful for selecting the optimal cutoff probability to guide decision making, as noted in the NCCN guidelines (version 4.2024) for *BRCA1/2* probability models.^7,8,10^ A meaningful output can also aid communications between health care providers and patients, which remain a bottleneck for rare diseases such as LFS.^6^

Our validation results also have important implications regarding clinical applications of risk prediction models in general. Given the discernible decrease in performance as we move from RPB to CCB, it is important for the research community to be aware of the differences between the two categories and, accordingly, dedicate new studies to truly CCB data sets^21-26^ to accurately evaluate the real-world performance of risk prediction models. The next steps to bring risk prediction models like LFSPRO closer to clinics should include a prospective evaluation of one CCB family at a time to further refine the picture of how risk prediction can transform clinical practice. Finally, the negative effects of missing data highlight an important question that is whether health care providers and patients can work together to improve data collection efficiency under the time constraints of clinical sessions.

## Data Availability

The conducted study has been approved by the institutional IRB at MD Anderson Cancer Center. The latest version of LFSPRO is publicly available on GitHub (https://github.com/wwylab/LFSPRO). The LFSPROShiny application is open-source on GitHub (https://github.com/wwylab/LFSPRO-ShinyApp) and is hosted live on Shinyapps.io (https://namhnguyen.shinyapps.io/lfspro-shinyapp-master/).
